# Obstetrics

**Published:** 1878-11

**Authors:** 


### Obstetrics.

Obliteration of the Uterine Neck in a Pregnant Woman. (*Journ. de Méd.* from *Annales de la Société Medico-Chirugicale de Liège.*)

M. Piron publishes an interesting case of a woman who had granular metritis after a first pregnancy. The local treatment consisted in cauterizations with solid silver nitrate, astringent injections, and applications of iodide of potassium in glycerine by means of a sponge, according to Scanzoni's method. It was during this treatment that the woman became pregnant a second time. The first stages of this pregnancy passed unnoticed on account of absence of sympathetic phenomena, and it was only after the belly began to grow that the fact was discovered, and the cauterizations were not discontinued until the sixth month. This circumstance of pregnancy developing in spite of the disease affecting the womb, and especially in spite of the means employed to combat it, gave rise to the fear that the inflammation of the neck, joined to the congestion produced by the pregnancy, might lead to obliteration of the neck. This indeed occurred. Some days before the abortion the touch proved the complete absence of the neck and uterine orifice. The speculum even did not permit the distinguishing of the slightest orifice, only a sort of furrow where it had formerly existed. A probe passed into this furrow could not penetrate to the uterine cavity. A few days after labor began the position could readily be made out through the thin wall of the womb, but no orifice was produced. M. Piron tried in vain to pass his nail through the groove he had seen. He decided to perform vaginal hysterotomy, and, by the aid of a speculum, he opened, with a bistoury, the uterus at the place of the neck, cutting from left to right. It was repeated in one hour, the first not being sufficient; but little blood was lost; the labor progressed slowly. After the neck was sufficiently dilated, the membranes were ruptured and forceps applied, a living child being extracted. The patient did well, and the neck remained permeable several months after the operation.

